# Pheochromocytoma/Paraganglioma Syndrome Type 1 Presenting with Atypical Symptoms and a Novel Pathogenic Variant in the *SDHD* Gene: A Case Report

**DOI:** 10.34172/aim.28810

**Published:** 2024-08-01

**Authors:** Elham Zohrehvand, Nastaran Injinari, Maryam Kiani Feyzabadi, Kazem Aghili, Farahnaz Ghaemi, Reyhaneh Azizi

**Affiliations:** ^1^University of Social Welfare and Rehabilitation Sciences, Tehran, Iran; ^2^Diabetes Research Center, Shahid Sadoughi University of Medical Sciences, Yazd, Iran; ^3^Cellular and Molecular Research Center, Basic Health Sciences Institute, Shahrekord University of Medical Sciences, Shahrekord, Iran; ^4^Department of Radiology, Shahid Sadoughi University of Medical Sciences, Yazd, Iran; ^5^Department of Biology, Kerman Branch, Islamic Azad University, Kerman, Iran

**Keywords:** Paraganglioma, Pediatric, SDHD, Whole exome sequencing

## Abstract

This case report presents a 10-year-old patient diagnosed with pheochromocytoma/paraganglioma syndrome type 1 (PPGL1), underlined by a novel heterozygous pathogenic variant (c.154_161del, p.ser52Profster14) in the *SDHD* gene. Initially, the patient manifested symptoms unusual for pheochromocytoma, including polyuria and polydipsia; however, further diagnostic investigations revealed a pheochromocytoma (PCC) tumor in the adrenal gland. Subsequently, whole exome sequencing (WES) test identified a pathogenic frameshift variant in the *SDHD* gene, strongly suggestive of PPGL1. This study highlights the importance of considering atypical symptoms in diagnosing rare pediatric pheochromocytoma/paraganglioma tumors and underscores the value of genetic testing in identifying underlying genetic causes, thereby facilitating personalized management of the condition.

## Introduction

 Paragangliomas (PGLs) are tumors derived from the neural crest chromaffin tissues that develop in the nerve tissue in the adrenal glands, along major blood vessels and nerve pathways in the neck and head area, and, less frequently, in other parts of the body.^1^ When this kind of tumor occurs in the adrenal glands, it is referred to as pheochromocytoma (PCC), which originates from chromaffin cells of the adrenal medulla and releases catecholamines, including epinephrine (EPI), norepinephrine (NE), and dopamine.^2^ Paraganglioma is considered to be a rare condition, with a prevalence that falls within the range of 0.2% to 0.6% among the general population.^3^ In children, the condition typically appears between the ages of 11 and 13, and there is a higher occurrence among males, with a ratio of 2 males to 1 female.^1^ Although the majority of patients with PCC and paraganglioma tumors are considered sporadic, about 30% of patients are found to have hereditary syndromes.^4^ Mutations in genes of the SDHx family, including *SDHA* [OMIM *600857], *SDHB* [OMIM *185470], *SDHC* [OMIM *602413], and *SDHD* [OMIM *602690] are primarily linked to the development of hereditary paraganglioma syndromes. Mutations in the *SDHD* gene lead to Familial Paraganglioma Syndrome type 1 (FPGL1) [OMIM#168000] with an autosomal dominant pattern of inheritance.^5^ Up to now, 185 unique DNA variants in the *SDHD* gene have been publicly reported, with an updated list available at: https://databases.lovd.nl/shared/genes/SDHD.^6^ This case report aims to present a 10-year-old patient diagnosed with PPGL1 with a novel mutation in the *SDHD* gene.

## Case Report

**Figure 1:**
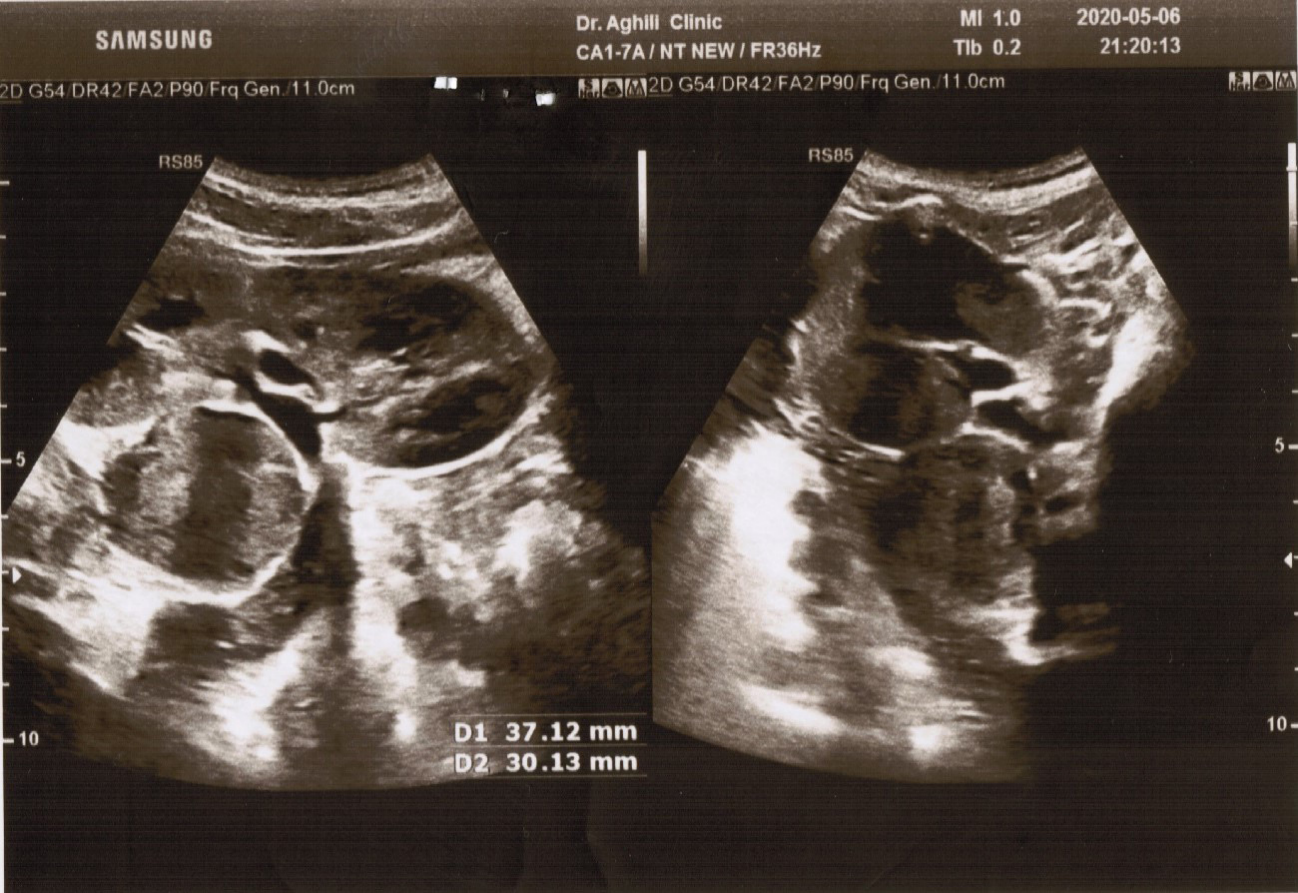
Abdominal Ultrasound Showing a Retroperitoneal Mass on the Medial Border of Left Kidney with a Diameter of 30 mm × 37 mm.

 A 10-year-old boy who presented with hypertension, polyuria, polydipsia, hyperhidrosis, and occasional outbursts of anger and aggression was admitted to our clinic. According to his parents’ assertion, he was producing more than 5 liters of urine per day, and several urine 24-hour volume tests confirmed similar amounts of urine production. The patient’s parents are healthy, and showing no clinically significant symptoms. Considering the normal level of fasting blood sugar and low urine-specific gravity in the patient’s urine tests, a water deprivation test was performed, which was negative, suggesting the possibility of primary polydipsia. The patient’s abdominal pain prompted the request for an abdominal ultrasound. The ultrasound study revealed a retroperitoneal mass on the medial border of the left kidney with a diameter of 30 mm × 37 mm (Figure 1). For further investigation, a chest spiral computerized tomography (CT) scan of the abdomen and pelvis was carried out. The study showed a heterogeneous enhancing soft tissue mass lesion measuring 33 mm × 27 mm in the lateral limb of the left adrenal gland that was suggestive of neoplastic lesions such as PCC. Moreover, magnetic resonance imaging (MRI) of the adrenal with and without contrast was performed, showing a well-defined heterogeneous lesion that was low in T1 and iso-signal in T2 with moderated contrast enhancement measuring 30 mm × 31 mm in the left adrenal gland, which could be a PCC or neoplastic lesion such as neuroblastoma. In the next step, comprehensive blood and urine laboratory tests were performed to determine if they correlated with the diagnostic imaging findings. Urine biochemical tests showed that the normetanephrine level was higher than twice the normal level, and vanillylmandelic acid was over 1.5 times the upper reference limit. However, homovanillic acid in urine was within the normal range, ruling out the possibility of neuroblastoma. Additionally, a metaiodobenzylguanidine (MIBG) scan was performed to investigate possible paraganglioma tumors, but no abnormalities were found in the study. A spiral CT scan showed no growths or nodules in the patient’s lungs, and echocardiography and eye examination results were normal. All the findings supported the necessity of surgical treatment. The patient was prescribed oral phenoxybenzamine for ten days before the laparoscopy operation in order to reduce the probability of the incidence of intraoperative hypertensive crises. Our case did not experience any sweating or hypertension during the procedure. The excised adrenal mass underwent pathological examination, which confirmed the diagnosis of PCC. Following the procedure, the patient did not display any noticeable clinical symptoms, and his hypertension was resolved. Subsequent blood examinations were carried out, revealing that all parameters were within the normal range. The biochemical analysis of urine, including vanillylmandelic acid, normetanephrine, and metanephrine levels, also showed that all parameters were within the reference range, and the 24-hour urine volume was normal. Furthermore, a whole exome sequencing (WES) test was conducted to determine the potential genetic factor behind the condition in our patient. The genomic DNA was extracted from the peripheral blood sample of the patient using the salting-out technique, and WES was executed utilizing the SureSelectXT Human All Exon V6 kit on the Illumina NextSeq500 platform. The interpretation and selection of variants were guided by the American College of Medical Genetics and Genomics (ACMG) standards for sequence variant analysis, incorporating data from databases and *in-silico* prediction tools such as gnomAD (https://gnomad.broadinstitute.org/), the 1000 Genomes Project (https://www.internationalgenome.org/), Online Mendelian Inheritance in Man (OMIM) (https://www.omim.org/), ClinVar (https://www.ncbi.nlm.nih.gov/clinvar/), MutationTaster (https://www.mutationtaster.org/), and Genomic Evolutionary Rate Profiling (GERP + + ) (http://mendel.stanford.edu/sidowlab/downloads/gerp/index.html). For further evaluation, we also checked out the candidate variants on the Iranome database as a local genomic repository.^7^

**Figure 2:**
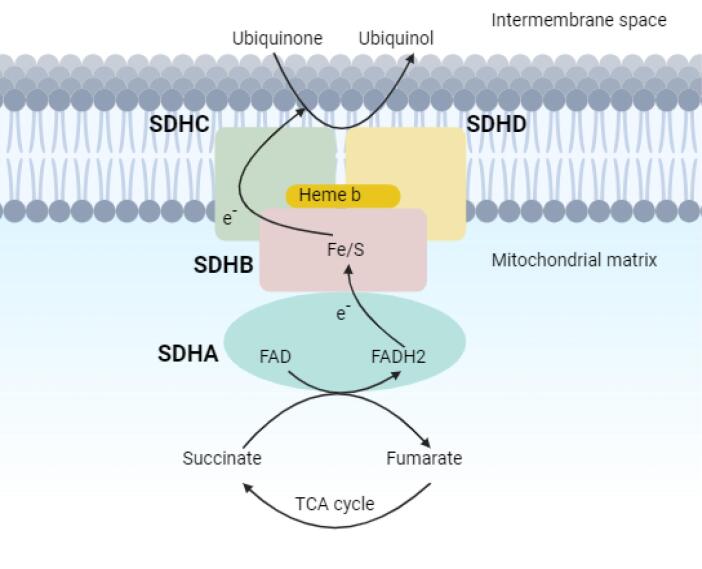
SDH, or Complex II, Crucial for the TCA Cycle and Aerobic Respiratory Chain, Catalyzing the Conversion of Succinate to Fumarate and the Reduction of Ubiquinone to Ubiquinol

**Figure 3:**
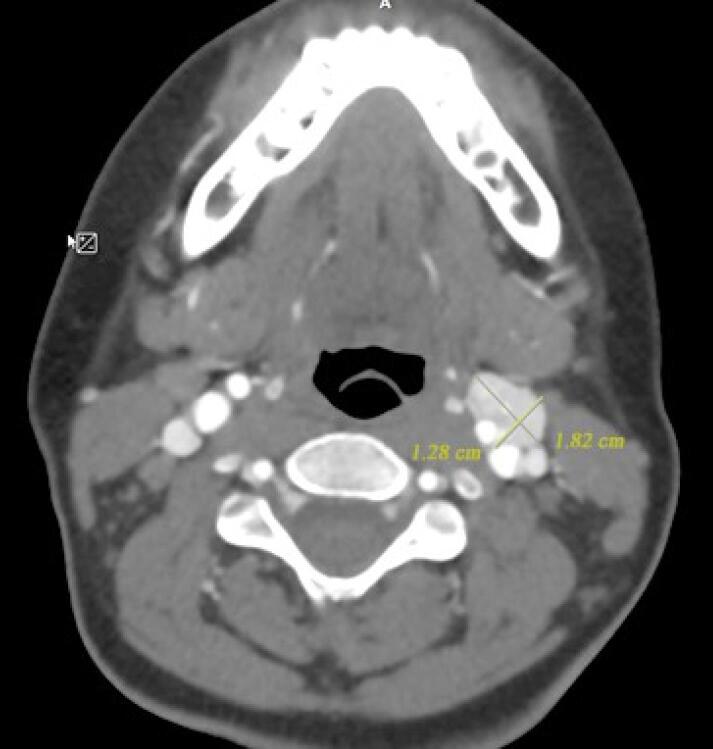
Multislice spiral CT Angiography Revealing an Avidly Enhancing Mass Lesion, Approximately 13 mm × 18 mm in Diameter

 Please refer to the Supplementary File 1 (Methods applied in WES test, including sample preparation, sequencing techniques, and data analysis approach for variant detection), and Supplementary File 2. The WES test revealed a frameshift mutation (Chr11:111958680:TGTCACCGA > T, NM_003002.4:exon2:c.154_161del:p.ser52Profster14) in the *SDHD* gene in our case. According to the ACMG guideline, the variant is categorized as pathogenic. Afterward, Sanger sequencing of the candidate variant in the patient confirmed the result of the WES study. Considering the possible inheritance of the disease, the patient’s parents underwent a Sanger sequencing test for the candidate variant, and it was not found in either parent, suggesting a *de novo* mutation. To our knowledge, this is a novel variant that is absent in all the population databases, and, at the time of writing this report, the variant has not been reported in any database or literature related to any human phenotype. Based on ACMG standards and guidelines, this variant is classified as pathogenic. The *SDHD* (Succinate Dehydrogenase Complex Subunit D) is a gene that encodes one of the four subunits of the respiratory complex II or succinate dehydrogenase (SDH) complex, also known as Cytochrome b. This subunit helps secure the SDH enzyme, which is an essential metabolic enzyme complex involved in generating ATP, within the membrane of the mitochondria. As shown in the Figure 2 the SDH enzyme plays a critical role in connecting the TCA cycle (tricarboxylic acid cycle) with oxidative phosphorylation, which are both vital pathways in energy conversion.^8^ Mutations in the *SDHD* gene result in PPGL1 syndrome, characterized by tumors mainly in the head and neck region, less frequently in the thoracoabdominal area, and the potential development of PCCs.^8^ Having the result of the WES test, comprehensive diagnostic imaging tests were performed to investigate other possible findings associated with the result of the genetic study. The patient underwent abdominal and pelvic MRI with and without contrast, spiral chest CT scan, and brain MRI. None of these tests revealed any abnormalities. However, a thyroid and neck soft tissue ultrasound study described an 11 mm × 18 mm lesion on the left side of the neck and at the bifurcation of the carotid artery, which was determined to be a carotid body tumor. In the ultrasound study, reactive lymph nodes were also observed on both sides of the neck, the largest of which was 23 mm × 5 mm on the right side. For further investigation, a multislice spiral CT angiography of the carotid arteries was conducted that revealed an avidly enhancing mass lesion between the left internal carotid and external carotid artery with anterior extension measures approximately 13 mm × 18 mm that could be a carotid body tumor (Figure 3). In the study, several bilateral anterior and posterior cervical chain lymph nodes with a maximum size of 15 mm × 9 mm were also seen. Moreover, in the soft tissue of the neck MRI, a 20 mm × 10 mm carotid body tumor and multiple reactive lymph nodes on both sides of the neck were detected. Since all of the investigations supported the diagnosis of a carotid body paraganglioma (CBP), our case underwent surgical resection of the tumor. Postoperative pathological assessment of the tumor confirmed the diagnosis of a CBP. Another finding in our case was a 12 mm cortical cyst in the upper pole of the left kidney detected in pelvic and abdominal ultrasound scans. Based on the comprehensive analyses, particularly the outcomes of the WES test, the patient was diagnosed with PCC/paraganglioma syndrome type 1.

## Discussion

 PGLs are rare tumors that arise from chromaffin cells. Prompt detection of this rare condition is vital, as it can pose life-threatening consequences if left undiagnosed.^1^ However, diagnosing this condition is often difficult because its symptoms resemble those of various other medical disorders, and determining an effective diagnostic approach, including laboratory tests and diagnostic imaging, can be confusing. Adding to this complexity is the need to comprehend the genetic basis of the tumors and approach each case based on the specific gene in which pathogenic mutations are identified. Our patient’s initial presentations were polyuria and polydipsia, which are not typical manifestations of PCC. However, subsequent imaging and laboratory tests, prompted by the patient’s abdominal pain, indicated a potential diagnosis of PCC, which was later confirmed through tissue pathological study. Following the therapeutic surgery, and since one significant indication to recommend a genetic test is when an individual under the age of 40 is diagnosed with PCC,^9^ the family was referred to genetic counseling. They were recommended to undergo a WES test to uncover any potential genetic factors contributing to the condition, as more than 30% of pediatric PCC cases may carry a germ-line mutation(s).^10^ Unfortunately, the COVID-19 pandemic resulted in the postponement of the genetic test. Finally, two years after the surgical treatment, the results of the patient’s WES study revealed a heterozygous pathogenic mutation in the *SDHD* gene. Taking the result of the genetic test into account and due to the increased likelihood of multiple tumors in individuals with mutations in the *SDHD* gene, as presented in Table 1, several ultrasound, MRI, and CT scan studies were conducted to detect any possible tumors in other parts of the body. These investigations led to the identification of a CBP, which is the most commonly occurring head and neck paraganglioma (H&N-PGL).^11^ Hypersecretion of catecholamines is extremely rare in patients with head-and-neck paragangliomas (HNPs), and the tumors are typically painless,^12^ which may explain the absence of abnormal laboratory test results, symptoms, or pain in our case after the removal of the PCC, despite the presence of HNP. Besides, pelvic and abdominal ultrasound scans showed a cortical cyst in the upper pole of the left kidney. Although this is not a neoplastic cyst, we will conduct follow-up imaging to monitor for any potential sign of malignant transformation. Our case further emphasizes the significance of genetic counseling and studies in children diagnosed with PCC and paraganglioma. It is also worth noting that although a MIBG scan conducted prior to the first therapeutic surgery did not identify any abnormalities, the subsequent diagnostic imaging tests conducted two years post-operation revealed the presence of paraganglioma tumors. This highlights the importance of repeating diagnostic imaging tests to detect any other extra-adrenal paragangliomas in patients diagnosed with PCC; it is particularly important in pediatric cases since recurrent tumors are more common in children with PGLs than in adults.^13^ We found a novel heterozygous mutation in the *SDHD* gene in a patient who had been showing unusual symptoms, and we will report any significant clinical manifestations that may arise in the future. According to the OMIM database, mutations in the gene are responsible for three different syndromes: Mitochondrial complex II deficiency, nuclear type 3; Paragangliomas and gastric stromal sarcoma; and PCC/paraganglioma syndrome type 1. Considering the symptoms manifested by our case, including hypertension, head and neck paragangliomas, PCC, and elevated catecholamines, PCC/paraganglioma syndrome type 1 was identified as the patient’s diagnosis. Moreover, the heterozygous state of the variant in our patient is consistent with the autosomal dominant pattern of inheritance of the syndrome according to the OMIM database. Furthermore, the patient exhibits additional symptoms, including polyuria, polydipsia, and hyperhidrosis, which are atypical initial indicators of the condition. This underscores the importance of recognizing and considering atypical symptoms in the diagnosis and management of PCC and paraganglioma syndromes.

**Table 1:** Frequency of Different Clinical Presentations Reported in Patients with Mutations in the *SDHD* Gene.

**Clinical Presentations**	**Frequency of Clinical Manifestations Related to Mutations in the ** ***SDHD***** Gene**
Head and neck paraganglioma (H&N-PGL)	50-70%
Thoracic-abdominal paraganglioma (TAP-PGL)	20-30%
Pheochromocytoma (PCC)	10-20%
Gastrointestinal stromal tumors (GIST)	5-10%
Pituitary neuroendocrine tumors (PNET)	5-10%
Renal cell carcinoma (RCC)	5-10%

## Conclusion

 In pediatric cases of PGLs, the clinical manifestations can be extremely variable; therefore, even rare symptoms should not be overlooked to ensure prompt diagnosis and treatment. Multidisciplinary approaches including laboratory and imaging tests should be used for an accurate diagnosis. Moreover, genetic tests could be helpful for diagnosis and managing the condition.

## Supplementary Files


Supplementary file 1 contains methods applied in WES test for variant detection

Supplementary file 2 contains variants identified for the patient
