# Supplementary material for: Pheochromocytoma/Paraganglioma Syndrome Type 1 Presenting with Atypical Symptoms and a Novel Pathogenic Variant in the SDHD Gene: A Case Report
Source: Arch Iran Med. 2024 Aug 1;27(8):447–51. doi: 10.34172/aim.28810 (PMC11416696; doi:10.34172/aim.28810)
Supplement: Supplementary file 2 — contains variants identified for the patient [file aim-27-447-s002.pdf]

Arch Iran Med. August 2024;27(8):447-S2

10.34172/aim.28810

Supplementary file 2

Supplementary Note

We performed whole exome sequencing (WES) to unravel the potential genetic underpinnings of the pheochromocytoma tumor in our case. Initially, a 10-mL blood sample was collected from the patient, using ethylenediaminetetraacetic acid (EDTA) as anticoagulant. The extraction of genomic DNA was then carried out through the salting-out technique, chosen for its ability to yield DNA of high quality, suitable for in-depth genetic analysis. Following DNA extraction, the exome enrichment was performed using SureSelect Human All Exon V6 from Agilent Technologies Inc. (Santa Clara, California), and sequencing of the enriched exome was undertaken on the Illumina NextSeq500 platform from Illumina Inc. (San Diego, California).

In the next step, a bioinformatics pipeline for analyzing the WES data was developed to precisely identify genetic variations. The methodology includes quality assessment using FastQC, read trimming and filtration with the NGS QC Toolkit, alignment to the reference genome GRCh37/hg19 via Burrows-Wheeler Aligner (BWA-MEM), duplicate marking and sorting using SAMtools and Picard Tools, and base quality score recalibration (BQSR) with GATK's BaseRecalibrator. Variant calling was performed with GATK's HaplotypeCaller, followed by variant quality score recalibration (VQSR) using GATK's VariantRecalibrator and ApplyVQSR tools to filter false positives. The result of this process was a VCF file containing 573,775 variants, ready for downstream analyses.

In the next step, variant annotation was performed using the ANNOVAR software. We then filtered out variants with a minor allele frequency (MAF) > 0.01, as cataloged in population databases such as the 1000 Genome Project, ExAC, and so forth, to filter out common variants. Then, prioritizing variants predicted to have a functional impact, we narrowed our focus to non-synonymous variants, including missense, nonsense, frameshift, splice site, and in-frame indels. At this step, we had 1,862 variants. In the next step, we filtered our data considering the available panel for pheochromocytoma and paraganglioma tumors containing 11 genes: *FH*, *MAX*, *NF1*, *RET*, *SDHA*, *SDHAF2*, *SDHB*, *SDHC*, *SDHD*, *TMEM127*, and *VHL*. Our patient had 97 variants in the mentioned genes. The assessment of these variants was further refined by consulting prediction scores from tools such as SIFT, PolyPhen-2, MutationTaster, MutationAssessor, and so forth, leading to the selection of 19 variants. Refer to Supplementary Table 1 for more details on the variants. The determination of causal variant(s) was further guided by genotype-phenotype correlations using databases such as OMIM and ClinVar, as well as resources like VarSome to follow ACMG guidelines. Finally, we reported the following variant as responsible for the phenotype in our patient: Chr11:111958680:TGTCACCGA>T, NM_003002.4:exon2:c.154_161del:p.Ser52ProfsTer14.
